# Evolutionary fate and implications of retrocopies in the African coelacanth genome

**DOI:** 10.1186/s12864-015-2178-9

**Published:** 2015-11-10

**Authors:** Kang Du, Shunping He

**Affiliations:** Key Laboratory of Aquatic Biodiversity and Conservation of the Chinese Academy of Sciences, Institute of Hydrobiology, Chinese Academy of Sciences, Wuhan, Hubei 430072 China; University of Chinese Academy of Sciences, Beijing, 100049 China

**Keywords:** Coelacanth | retrocopy | novel genetic elements

## Abstract

**Background:**

The coelacanth is known as a “living fossil” because of its morphological resemblance to its fossil ancestors. Thus, it serves as a useful model that provides insight into the fish that first walked on land. Retrocopies are a type of novel genetic element that are likely to contribute to genome or phenotype innovations. Thus, investigating retrocopies in the coelacanth genome can determine the role of retrocopies in coelacanth genome innovations and perhaps even water-to-land adaptations.

**Results:**

We determined the dS values, dN/dS ratios, expression patterns, and enrichment of functional categories for 472 retrocopies in the African coelacanth genome. Of the retrocopies, 85–355 were shown to be potentially functional (i.e., retrogenes). The distribution of retrocopies based on their dS values revealed a burst pattern of young retrocopies in the genome. The retrocopy birth pattern was shown to be more similar to that in tetrapods than ray-finned fish, which indicates a genomic transformation that accompanied vertebrate evolution from water to land. Among these retrocopies, retrogenes were more prevalent in old than young retrocopies, which indicates that most retrocopies may have been eliminated during evolution, even though some retrocopies survived, attained biological function as retrogenes, and became old. Transcriptome data revealed that many retrocopies showed a biased expression pattern in the testis, although the expression was not specifically associated with a particular retrocopy age range. We identified 225 Ensembl genes that overlapped with the coelacanth genome retrocopies. GO enrichment analysis revealed different overrepresented GO (gene ontology) terms between these “retrocopy-overlapped genes” and the retrocopy parent genes, which indicates potential genomic functional organization produced by retrotranspositions. Among the 225 retrocopy-overlapped genes, we also identified 46 that were coelacanth-specific, which could represent a potential molecular basis for coelacanth evolution.

**Conclusions:**

Our study identified 472 retrocopies in the coelacanth genome. Sequence analysis of these retrocopies and their parent genes, transcriptome data, and GO annotation information revealed novel insight about the potential role of genomic retrocopies in coelacanth evolution and vertebrate adaptations during the evolutionary transition from water to land.

**Electronic supplementary material:**

The online version of this article (doi:10.1186/s12864-015-2178-9) contains supplementary material, which is available to authorized users.

## Background

Genes that are unnecessary for existence in a new environment are often eliminated during the course of evolution, but genomes also acquire novel genetic elements as a source of functional and phenotypic diversity [1]. Retrocopies, one type of novel genetic element, are genome segments that are reverse-transcribed from intronless mRNA and then inserted into new positions in the genome (i.e., RNA-based duplication) [1]. Retrocopies have long been considered evolutionary dead ends, because it was expected that these segments lack regulatory elements and originate from RNA-based duplication [2, 3]. However, many recent studies revealed that retrocopies can be used to generate new genes, called retrogenes, by fusion with other genes [4–6] or via acquisition of new exons or introns from *de novo* genome sequences [4]. Furthermore, retrogenes, which are functional retrocopies, have often evolved functional roles in male germ lines [7, 8]. Thus, “dead on arrival” copies of parental genes are currently considered to serve as “seeds of evolution” [9]. Despite the substantial contribution to molecular evolution revealed by studies on retrocopies in humans and fruit flies [10–13], the patterns of retrocopy formation and effects on genomic dynamics of lower vertebrates remain undetermined.

The African coelacanth is the second closest living fish relative of tetrapods. Thus, genomic data on this species, which have recently been made available, are useful for researching successful land adaptations during vertebrate evolution [14]. The African coelacanth is well known for its morphological resemblance to its fossil ancestors; as anticipated, protein-coding sequence evolution in the coelacanth genome was shown to be relatively slow [14]. Because retrogenes can serve as “seeds of evolution,” investigation into the pattern and rate of retrogene formation and implications of the role of these retrogenes in the African coelacanth genome can provide novel insight into coelacanth evolution and water-to-land vertebrate adaptations.

In this study, we identified and investigated 472 parent-retrocopy pairs in the African coelacanth genome. Based on the obtained results, we suggest that retrocopies attained biological relevance and were subject to natural selection following fusion with existing genes or acquisition of new gene elements, although many retrocopies have ultimately been eliminated from the genome during coelacanth evolution. Analysis of these potentially functional retrocopies (or retrogenes) provides new insight into the dynamics of functional organization of the genome during coelacanth evolution and vertebrate transition from water to land.

## Results

### Retrocopies and potential retrogenes in the African coelacanth genome

Although retrocopies were long thought to be pseudogenes, it is now known that many are functional and considered retrogenes following fusion with other genes [5] or acquisition of regulatory elements and exons from *de novo* sequences [4, 15]. To evaluate whether a retrocopy is functional, multiple criteria, such as a dN/dS ratio <0.5 or transcriptional evidence, were developed in previous studies [13, 16–18].

**Fig. 1:**
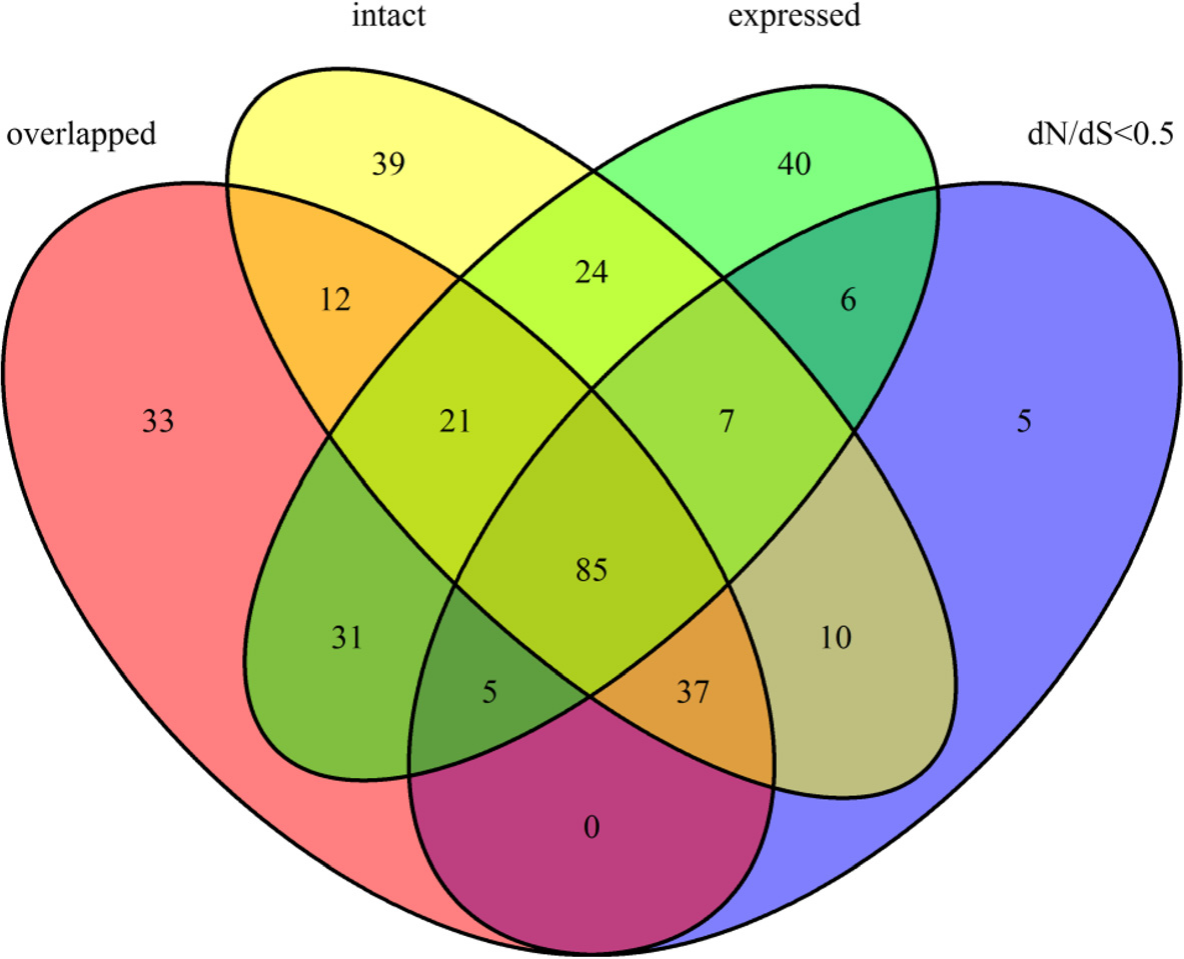
Venn diagram of the unique and common retrocopies among different categories. Red refers to retrocopies that overlap with Ensembl genes. Yellow refers to retrocopies with intact ORFs. Green refers to retrocopies with evidence of transcription. Blue refers to retrocopies with dN/dS ratios significantly <0.5

In this study, 472 one-to-one parent-retrocopy pairs were identified in the African coelacanth genome (Additional file 1: Table S1). To assess retrocopy functionality, we first compared the sequences of each retrocopy and its parent gene, which revealed a total of 235 intact retrocopies with no frameshift mutations or premature stop codons (Fig. 1, Additional file 1: Table S1). These retrocopies possess intact ORFs (open reading frames) and thus have the potential to become functional protein sequences [19]. Additionally, 155 retrocopies with a dN/dS ratio significantly <0.5 were also identified (Fig. 1, Additional file 1: Table S1). dN/dS <0.5 is indicative of purifying selection, which reflects potential for retrocopy functionality [13]. Second, we mapped retrocopies to Ensembl genes (http://www.ensembl.org) to detect retrocopies showed overlap with Ensembl genes. In this step, 224 retrocopies were detected, which included 152 exon-overlapping retrocopies and 72 non-exon-overlapping retrocopies (Fig. 1, Additional file 1: Table S2). These retrocopies may represent the main direct way by which retrocopies can influnce the genome, fusing with an existing gene, or acquiring new gene elements from a *de novo* sequence [4, 15, 20]. Finally, we mapped the RNA-seq data (gills, kidneys, pectoral fins, pelvic fins, pharynx, tail muscle, and testis; SRA (sequence read archive) accessions DRP000627 and SRX189186; see Methods) to the African coelacanth genome and identified 219 retrocopies with transcriptional evidence (i.e., retrocopies with FPKM (fragments per kilobaseof exon per million fragments mapped) values, Additional file 1: Table S3).

The common and unique retrocopies identified by the previously discussed different criteria are summarized in Fig. 1. Pearson Chi-square tests revealed whether an expressed retrocopy (i.e., based on FPKM value) was intact, showed a dN/dS value <0.5, or overlapped with an Ensembl gene (*p*-values <0.001 respectively). These results indicate that the analyzed retrocopies met the criteria for functional retrocopies. Moreover, 355 retrocopies were identified by at least one of the four criteria, and 85 retrocopies were identified by all criteria; this indicates that 85–355 retrocopies are potential retrogenes (Fig. 1).

### Retrocopy age distribution

**Fig. 2:**
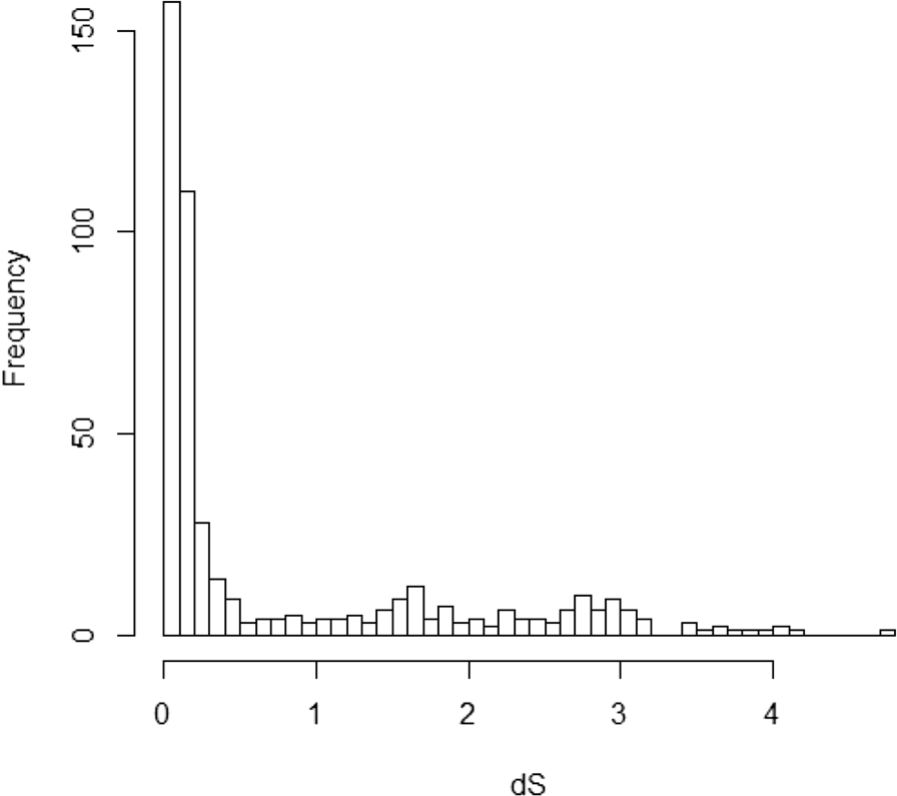
The dS distribution of the 472 retrocopies in the genome of the African coelacanth

**Fig. 3:**
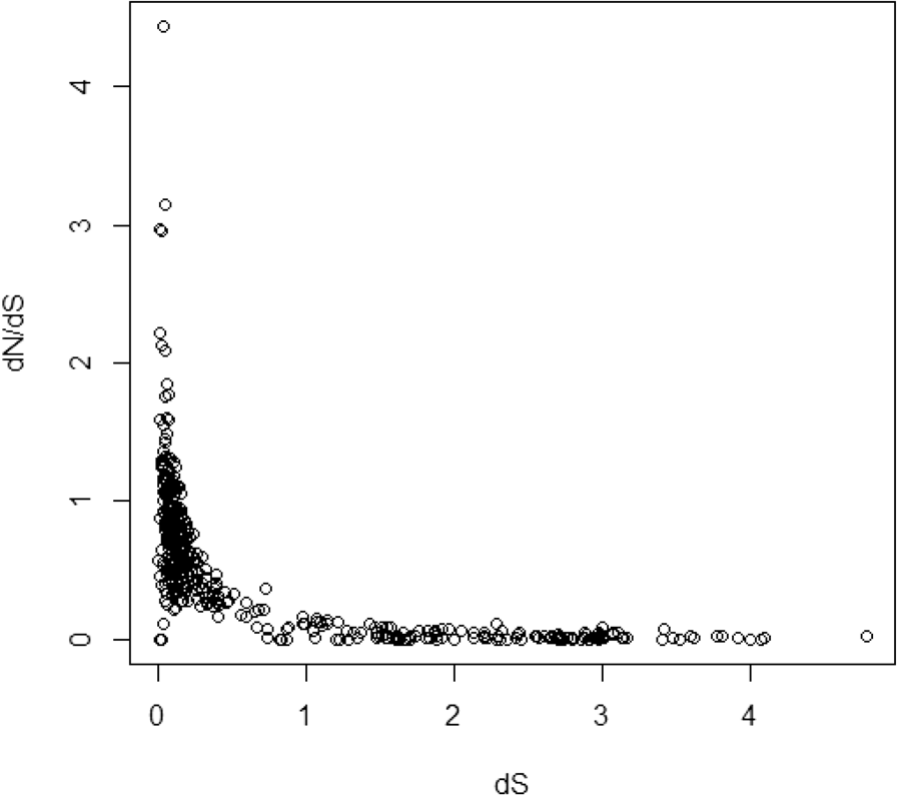
Dot plot of the dN/dS ratios with the dS values for each of the 472 retrocopies

To investigate the origin and evolution of these retrocopies, we assessed the retrocopy age distribution based on the increase in dS values, which were estimated by comparing the parent genes and retrocopies. The distribution revealed a burst pattern of young retrocopies (dS <0.6; Fig. 2). Notably, similar age distribution patterns of retrocopies were revealed in humans, platypuses, and western clawed frogs in a previous study [21]. However, this pattern was not generated in ray-finned fish, including sticklebacks, zebrafish, medaka, tetraodon, and fugu [21]. Moreover, the associated dN/dS ratios with the retrocopies tended to decrease as dS increased (Fig. 3), which indicates a greater constraint of selection on older retrocopies.

**Fig. 4:**
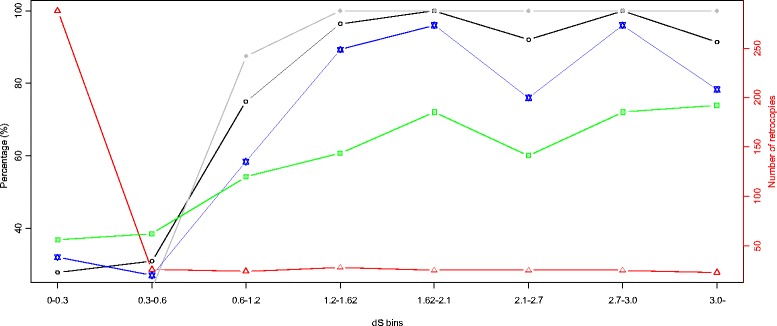
Percentages of retrocopies with different indications of functionality in each dS bin, distributed according to the increase in dS values. The red line corresponds to the right Y-axis and represents the number of retrocopies. The lines in black, grey, blue and green correspond to the left Y-axis and represent the percentages of retrocopies showing intact ORFs, dN/dS ratios significantly <0.5,overlap with Ensembl genes and transcriptional evidence, respectively

**Fig. 5:**
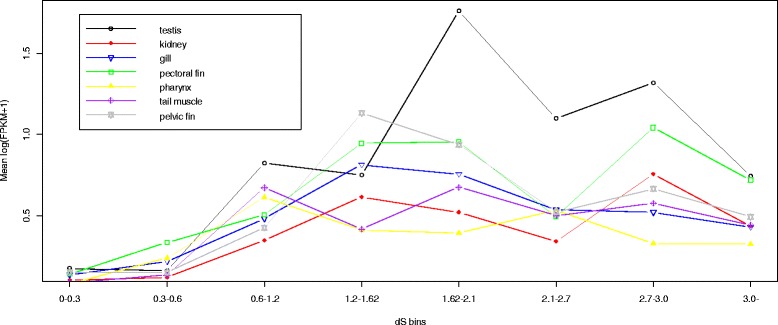
Mean expression levels of retrocopies in different dS bins. Expression levels were measured as log(FPKM + 1) values

**Fig. 6:**
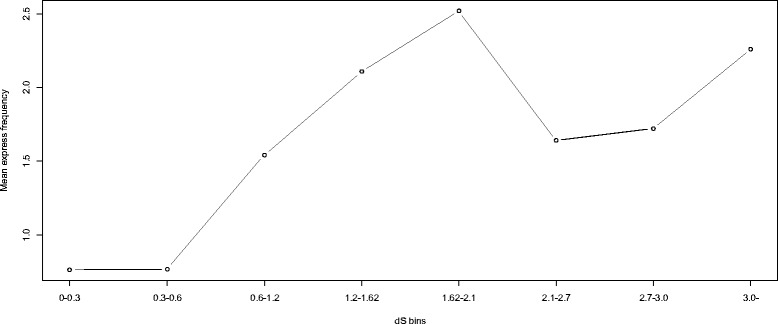
Mean expression frequency for retrocopies in different dS bins

To assess the proportions of retrogenes with different age ranks, we subdivided the retrocopies into bins based on dS intervals and then estimated the percentage of functional retrocopies for each bin. Regardless of the frequency burst of young retrocopies, the retrocopy bins with lower dS values (i.e., younger retrocopies) tended to contain a smaller percentage of retrocopies that exhibited intact ORFs, dN/dS ratios significantly <0.5, overlap with Ensembl genes, or expression compared with those that had elevated dS values (i.e., potentially functional retrocopies) (Fig. 4). Additionally, estimation of the mean log_10_(FPKM + 1) values for the retrocopies in each dS bin showed that elevated mean values tended to be associated with relatively old retrocopies (i.e., dS >0.6; Fig. 5). Finally, the “expression frequency” was considered the number of tissues that expressed a specific retrocopy, and an elevated expression frequency tended to be associated with relatively old retrocopies (Fig. 6).

### Expression patterns

A total of 219 retrocopies showed evidence of expression when we mapped the RNA-seq data to the African coelacanth genome. (Additional file 1: Table S3). In this study, we refer to the “expression frequency” as the number of tissues showing expression of a retrocopy; retrocopies with higher expression frequencies (frequency >3) tended to exhibit higher log(FPKM + 1) values for each tissue (*p* <0.01 Kruskal–Wallis test) and higher dS values (*p* <0.01 Kruskal–Wallis test), but lower dN/dS ratios (*p* <0.01 Kruskal–Wallis test) than the retrocopies with lower expression frequencies (Table 1). Young retrocopies were more likely than old retrocopies to be expressed with lower FPKM values and lower expression frequencies (Figs. 5 and 6).

**Table 1:** Mean and median value for dS, dN/dS and log(FPKM + 1) of retrocopies with different express frequency

Express frequency	number	mean log(FPKM + 1)^a^	median log(FPKM + 1)	mean dS	median dS	mean dN/dS	media dN/dS
0	250	0.000	0.000	0.518	0.147	0.629	0.577
1	100	1.397	0.965	0.939	0.172	0.482	0.367
2	36	1.430	0.692	0.953	0.328	0.472	0.358
3	25	1.191	0.618	0.832	0.161	0.508	0.556
4	12	2.116	2.222	1.659	1.649	0.165	0.012
5	16	1.763	1.835	1.734	1.776	0.281	0.016
6	15	1.924	1.031	1.600	1.770	0.204	0.060
7	10	3.340	3.016	1.185	1.046	0.354	0.017

^a^log(FPKM + 1) per retrocopy per tissue

In previous studies that examined retrocopies in humans and fruit flies [13, 22, 23], the preferential retrogene expression showed bias in the testis. In this study, 139 of the 472 retrocopies were expressed in the testis and were the most overrepresented retrocopies expressed in tissues (Additional file 1: Table S4). Additionally, the retrocopies expressed in the testis was also the most overrepresented of retrocopies that were uniquely expressed in one tissue (Additional file 1: Table S5), which indicates that this expression pattern bias also occurs in the coelacanth. However, despite the overrepresentation of retrocopy expression in the testis, no dS value or dN/dS ratio bias was detected in the retrocopy expression patterns in any tissue (Additional file 1: Tables S4 and S5).

### Retrocopy-overlapped genes

The primary mechanisms by which a retrocopy can be transcribed and become functional are fusion (or insertion) with an existing gene or acquisition of new gene elements from a *de novo* sequence [4, 15, 20]. In this study, 224 retrocopies overlapped with 225 coelacanth Ensembl genes. We defined the 225 Ensembl genes as “retrocopy-overlapped genes” (Additional file 1: Table S2), and these Ensembl genes might harbor inserted retrocopies or have originated from the *de novo* exon/intron acquisition of retrocopies (Additional file 2: Figure S1). These retrocopy-overlapped genes (i.e., Ensembl genes that overlapped with retrocopies) might represent the most direct pathway for retrocopies to influence the genome dynamics of the coelacanth. To further examine this direct influence, we compared the GO enrichment results for the retrocopy parent genes with those of the 225 retrocopy-overlapped genes. The results revealed that the gene functions that were overrepresented in the parent genes were most likely related to the synthesis and metabolism of biological molecules, whereas those in the retrocopy-overlapped genes were most likely related to transmembrane transport (Table 2). This result might indicate functional organization of the genome during coelacanth evolution, which was most likely produced by retrotransposition.

**Table 2:** Comparison of over-represented GO terms between parent genes of the retrocopies and the retrocopy-overlapped genes

	Category	GO.ID	Term	Annotated	Significant	Expected	classicFisher
Parent gene	BP	GO:0006278	RNA-dependent DNA replication	241	24	5.69	2.20E-09
Parent gene	BP	GO:0015074	DNA integration	76	13	1.8	2.10E-08
Parent gene	BP	GO:0006260	DNA replication	314	25	7.42	9.30E-08
Parent gene	BP	GO:0006259	DNA metabolic process	555	32	13.11	2.30E-06
Parent gene	BP	GO:0044260	cellular macromolecule metabolic process	3332	103	78.72	0.0005
Parent gene	BP	GO:0034645	cellular macromolecule biosynthetic process	1763	62	41.65	0.00051
Parent gene	BP	GO:0009059	macromolecule biosynthetic process	1772	62	41.86	0.00058
Parent gene	BP	GO:0044237	cellular metabolic process	4497	130	106.24	0.00092
Parent gene	BP	GO:0044267	cellular protein metabolic process	1574	55	37.19	0.00136
Parent gene	BP	GO:0044249	cellular biosynthetic process	2068	66	48.86	0.00419
Parent gene	BP	GO:1901576	organic substance biosynthetic process	2108	66	49.8	0.00663
Parent gene	BP	GO:0043170	macromolecule metabolic process	3799	108	89.75	0.00792
Retrocopy-overlapped gene	BP	GO:0006811	ion transport	592	21	6.84	3.40E-06
Retrocopy-overlapped gene	BP	GO:0044765	single-organism transport	1074	27	12.41	6.70E-05
Retrocopy-overlapped gene	BP	GO:0006810	transport	1617	34	18.68	0.00023
Retrocopy-overlapped gene	BP	GO:0051234	establishment of localization	1631	34	18.85	0.00027
Parent gene	CC	GO:0005840	ribosome	151	9	3.34	0.0061
Parent gene	CC	GO:0005737	cytoplasm	1294	41	28.61	0.0081
Parent gene	CC	GO:0044456	synapse part	83	6	1.83	0.0099
Retrocopy-overlapped gene	CC	GO:0044425	membrane part	2692	43	31.71	0.0073
Retrocopy-overlapped gene	CC	GO:0016021	integral component of membrane	2478	40	29.19	0.0091
Parent gene	MF	GO:0003676	nucleic acid binding	2867	158	77.88	1.50E-21
Parent gene	MF	GO:1901363	heterocyclic compound binding	4595	202	124.81	6.60E-17
Parent gene	MF	GO:0097159	organic cyclic compound binding	4608	202	125.17	9.30E-17
Parent gene	MF	GO:0046983	protein dimerization activity	419	35	11.38	3.40E-09
Parent gene	MF	GO:0003964	RNA-directed DNA polymerase activity	241	24	6.55	4.20E-08
Parent gene	MF	GO:0034061	DNA polymerase activity	262	25	7.12	5.00E-08
Parent gene	MF	GO:0003723	RNA binding	528	36	14.34	3.60E-07
Parent gene	MF	GO:0016779	nucleotidyltransferase activity	319	25	8.67	2.10E-06
Parent gene	MF	GO:0005488	binding	10088	298	274.02	0.002
Parent gene	MF	GO:0016772	transferase activity, transferring phosphorus-containing groups	1051	43	28.55	0.0045
Retrocopy-overlapped gene	MF	GO:0015075	ion transmembrane transporter activity	594	22	8.11	2.00E-05
Retrocopy-overlapped gene	MF	GO:0022891	substrate-specific transmembrane transport	623	22	8.51	4.20E-05
Retrocopy-overlapped gene	MF	GO:0022892	substrate-specific transporter activity	688	23	9.39	6.40E-05
Retrocopy-overlapped gene	MF	GO:0022857	transmembrane transporter activity	706	23	9.64	9.50E-05
Retrocopy-overlapped gene	MF	GO:0008324	cation transmembrane transporter activity	426	16	5.82	0.00025
Retrocopy-overlapped gene	MF	GO:0005215	transporter activity	877	25	11.98	0.00037

To further determine retrocopy influence on coelacanth and lower vertebrate evolution, we investigated potential orthologous sequences of the retrocopy-overlapped genes in ray-finned fish, including elephant sharks, fugu, zebrafish, gar, and some tetrapods, such as clawed frogs, anole lizards, zebra finches, mice, and humans. In particular, we focused on two retrocopy groups: those that were coelacanth-specific and those that were lost in tetrapods.

A total of 46 retrocopy-overlapped genes were identified as new retrogenes or lineage-specific genes for the coelacanth, and no orthologous sequence was found in any of the species investigated. These new genes most likely originated recently through the *de novo* acquisition of gene elements from retrocopies and not from host gene-retrocopy fusion. Thus, within the approximately 400 MY coelacanth evolutionary history [24], the rate of new retrogene formation has been relatively slow compared with that in humans (46/400 vs. 57/63, respectively) [13]. This result is consistent with the morphological resemblance and slow pace of evolution of protein-coding sequences in coelacanth genome.

Comparison of new retrogenes with the remainder of retrocopy-overlapped genes showed that these genes tended to contain fewer exons, were more likely overlap at an exon site, and were more underexpressed (Additional file 1: Table S6). Additionally, the retrocopies that were mapped to these genes tended to be younger, have a lower expression frequency, and were less constrained by natural selection (Additional file 1: Table S6). The GO analysis indicated that the overrepresented functional categories were most likely related to cell response, modification of biomolecules, and the immune function (Additional file 1: Table S7).

In addition to the 46 coelacanth-specific retrocopy-overlapped genes, we identified 23 genes that were specifically lost in tetrapods (there were orthologous sequences in the fish but not the tetrapods; Additional file 1: Table S8). This indicates that these genes might be unnecessary for living on land. In previous studies on these genes using zebrafish data (gene expression, knockdown, and knockout), some associations with important developmental categories were reported (Additional file 1: Table S8), including caudal fin development, eye morphology, vasculature development, and nervous system development [25]. Compared with the retrocopy-overlapped genes that still exist in tetrapod lineages, the 23 specifically lost genes did not show any difference in genetic structure or expression. However, the “lost” retrocopy-overlapped genes had lower dN/dS values than those that were retained, which indicates different selection pressures (Additional file 1: Table S9). Comparison of the GO enrichment results showed that the overrepresented functional categories for the lost genes were most likely to be related to the nervous system, whereas those in the “retained” gene set were most likely related to circadian rhythms and ionic transmembrane transport (Additional file 1: Table S10).

## Discussion

We explored the evolutionary fate and implications of retrocopies in the coelacanth genome and provided a novel perspective to understand the evolution of lower vertebrates and their adaptations in the transition from water to land. In total, we screened 472 retrocopies in the African coelacanth genome. The age distribution of the retrocopies based on obtained dS values showed a burst pattern of young retrocopies that accumulated in the genome (Fig. 1). Notably, similar patterns for retrocopy age distribution were found in humans, platypuses, and western clawed frogs, but not ray-finned fish [21]. This finding shows that the coelacanth’s birth pattern of retrocopies is more similar to that of tetrapods than that of ray-finned fish, which indicates that this pattern change might be related to the vertebrate evolutionary transition from water to land.

Because retropositions require RNA as a mediator, reverse transcriptase that stems from retrotransposons might be also necessary for retropositions. Reverse transcriptase encoded by long interspersed nuclear elements (LINEs), which possess endonucleolytic activity that can recognize any polyadenylated mRNA, seems to be responsible for retrotranspositions in mammals [1]. Additionally, as previously demonstrated [26, 27], the LINE-1 (L1) element subfamily of LINEs can generate processed genes, which indicates that L1 retrotransposon activity has generated retroposed gene copies in mammals [1]. As in non-mammalian chordates, it was also reported that the number of retrocopies correlated with the number of L1 copies, but not with any other type of LINE element, such as L2 [21]. Additionally, the difference between retrocopy birth pattern ray-finned fish and that in tetrapods coincides with the dramatic difference in L1 retrotransposon diversity between mammals and fish [28]. These results indicate that L1 may also be responsible for the retrotranspositions in non-mammalian chordates and represent a genetic signal that reflects the vertebrate evolutionary transition from water to land.

Despite the burst pattern of young retrocopies that accumulated in the genome, retrocopies with indications of function (dN/dS significantly <0.5, overlap with Ensembl genes, or evidence of transcription) were much more overrepresented among the old than young retrocopies (Figs. 4, 5 and 6). The overrepresentation of the retrogenes among the old retrocopies and decrease in frequency of relatively old retrocopies indicated that many of the retrocopies might have been eliminated during evolution, which was consistent with our predictions based on the expected lack of regulatory elements and introns in the retrocopies. Such features clearly indicate a course of rapid diversity within the evolutionary fate of the retrocopies before their elimination or incorporation into a pathway. However, the retrocopies could be affected by natural selection associated with the host genes in which the retrocopies are inserted, or the retrocopies could be constrained by natural selection after the acquisition of exons/new regulatory elements (Fig. 3). Such retrocopies might attain biological relevance and become evolutionarily stable and, thus, ultimately be overrepresented as other unstable retrocopies are eliminated. This hypothesis was also supported by our analysis of retrocopy-overlapped genes. The newly originated coelacanth-specific retrocopy-overlapped genes were younger than the other retrocopy-overlapped genes. As indicated by their lower expression level (Additional file 1: Table S6), the younger genes formed from the retrocopies might be less evolutionarily stable compared with the other retrocopy-overlapped genes. Moreover, these newly formed genes tended to contain fewer exons (Additional file 1: Table S6), which indicated that the new retrogenes might continue to recruit exons or introns during their course of evolution, or, as a complementary mechanism, these retrocopies might be preferentially inserted into host genes with more exons.

As previously reported, the retrogenes might be preferentially expressed in the testis. In this study, more retrocopies were expressed in the testis than in other tissues. However, no dS or dN/dS bias, which reflects retrocopy age range, was associated with any tissue-specific retrocopies (Additional file 1: Tables S4 and S5). Moreover, expression pattern analysis revealed that older retrocopies tended to be expressed with higher FPKM values and in more tissues than the young retrocopies (Fig. 6 and Table 1). The association of increased retrocopy expression distribution among tissues with increased age indicated that retrocopy biological functions might evolve to become essential over time [29].

The most direct ways in which a retrocopy can affect the genome are through retrocopy insertion into a gene and acquisition of new gene elements by the retrocopy. We identified 225 retrocopy-overlapped genes. GO enrichment analysis revealed that the overrepresented GO terms differed between the 225 retrocopy-overlapped genes and the parent genes from which those retrocopies originated. The gene functions that were overrepresented in the parent genes were most likely related to the synthesis and metabolism of biological molecules, whereas those of the 225 retrocopy-overlapped genes were most likely related to transmembrane transport. By retrotransposition of a father gene’s mRNA copy insertion into a gene, these father genes may contribute to the evolution of retrocopy-overlapped genes, whose functional organization differs from that of the father’s gene. This result indicates that retrotranspositions may contribute to functional organization dynamics of the genome.

Among the 225 retrocopy-overlapped genes, 23 genes that were specifically lost in the tetrapod lineage were identified, including *myh10*, which is related to eye morphology; *NTN1*, which is related to olfactory processes; and *esrrga*, which is related to caudal fin malformations (Additional file 1: Table S8). Comparisons between these genes and the retrocopy-overlapped genes with orthologous sequences in all of the investigated tetrapods revealed no differences in gene structure or expression level; however, as predicted from the retrocopies with which they overlapped, these lost genes tended to be more constrained by natural selection in the coelacanth genome. This result indicates that the genes were not lost in the tetrapod lineage because of any structural defect, but because during the adaptation of vertebrates from water to land, these genes were not necessary. Furthermore, the overrepresentation of functional categories related to the nervous system in these genes might indicate that, unlike the system of ionic homeostasis regulation (Additional file 1: Table S10), the sensory system of tetrapods may have experienced remodeling, which modified it from the system observed in fish.

The birth of new genes can contribute to the formation of lineage- or species-specific genes [30, 31], as found in this study. We identified 46 retrocopy-overlapped genes that were specific to coelacanths. The overrepresented gene functions related to the immune function indicated that the immune system was reinforced during coelacanth evolution (Additional file 1: Table S7). Additionally, the slow rate of new retrogene formation corresponded to the slow evolution of protein-coding genes in the coelacanth genome [14], which provided novel insight into the morphological resemblance of the coelacanth to its fossil ancestors. Together, these results might indicate stability of the deep sea environment in which the coelacanth evolved.

## Conclusions

Our study revealed a burst pattern of young retrocopies in the coelacanth genome. This pattern is similar to that in tetrapods rather than that in ray-finned fish, which indicates a possible genomic change related to water-to-land adaptations. Many retrocopies may have been eliminated during coelacanth genome evolution because of disrupted genetic structure defects. However, some might have been randomly inserted into existing genes or acquired regulatory elements, exons, or introns from *de novo* genetic sequences that facilitated overcoming of these defects and acquiring functions (i.e., to become retrogenes). These retrogenes were revealed to have an effect on functional organization of the genome, which provides novel insight into coelacanth evolution and the transformations involved in the transition from water to land. However, the results of this study were only sufficient for revealing some of the effects of retrotransposition on the genome; the response of the host gene to retrocopy insertion remains obscure, and retrocopies may function as noncoding RNAs that do not demand protein coding gene’s structures (such as ORF) [32]. Thus, our studies in the future will further explore these issues.

## Methods

### Retrocopy screening

First, to obtain potential single-exon ORFs in the Africa coelacanth (*Latimeria chalumnae*) genome scaffold as candidate retrocopies, we mapped all of the peptide sequences that were retrieved from Ensembl (http://www.ensembl.org) to the repeat-masked genome sequence (Ensembl release 78) using tBLASTn [33]. Then, a series of steps was applied to the tBLASTn output to screen for reliable results: 1) when the distance between the hits was <40 bp, adjacent homology hits were merged; 2) merged hit sequences with aligned regions of >50 amino acids and an amino acid identity >30 % were selected; and 3) among the overlapped target sequences, the longest sequence was selected. Second, we performed searches for similarity of the merged hit sequences against all peptide sequences (including single-exon proteins, to identify DNA-based duplication of intron-containing genes [34]) using FASTA. The multiple-exon peptide sequences with the closet hits were selected for subsequent pairwise comparisons in GENEWISE [35]. Before the analysis, the hit sequences were expanded by 10,000 bp on each flank. Finally, based on the GENEWISE results, any retrocopy candidates showing alignments of ≤50 amino acids, an amino acid identity ≤70 % or multiple exons were first excluded, after which we confirmed the absence of at least two intros in the retrocopy candidates. Finally, parent-retro pairs with a common parent peptide sequence were not included in our analyses.

### dN and dS estimation and dN/dS ratio test

The dN (nonsynonymous substitutions) and dS (synonymous substitutions) values were estimated for each retrocopy with its parent using the YN00 program of PAML4.8 [36]. To evaluate whether the dN/dS ratio between parent-retro pairs was significantly different from 0.5, we conducted a likelihood ratio test (LRT) using the codeml program of PAML4.8 in a pair-wise model. In the test, the null model was run with a fixed dN/dS = 0.5, and the alternative model was run to estimate dN/dS. Multiple testing was corrected via the false discovery rate method (FDR) [37] in R (http://www.R-project.org).

### Mapping to the Ensembl annotation

We mapped all of the retrocopies to Ensembl (release 79) genes using coordinate information. A gene that was mapped with a retrocopy was referred to as a “retrocopy-overlapped gene”. For each retrocopy that mapped to a gene, we regarded the retrocopy as an exon-overlapped retrocopy when it overlapped with any exon of that gene. Otherwise, the retrocopy was regarded as a non-exon-overlapped retrocopy.

### Gene expression analysis

Paired-end RNA-seq data from *Latimeria chalumnae* tissues, including the gills, kidneys, pectoral fins, pelvic fins, pharynx and tail muscle, were obtained from SRA accession DRP000627. Because of the 99.73 % identity between the testis transcriptome of *L. menadoensis* and the genome of *L. chalumnae* [14], we also included the RNA-seq data from the *L. menadoensis* testis (SRX189186). These reads were aligned against the African coelacanth annotated genome sequences using TopHat-2.0.13 [38] with a “--max-multihits 1” setting, which searched for the distinct best hit for each read. We estimated expression abundances using Cufflinks-2.2.1 [38] and measured the abundances in FPKM (fragments per kilobase of transcript per million fragments mapped). Both programs were run with the default settings.

### Gene ontology (GO) analysis

The GO annotations for the African coelacanth were downloaded from the Ensembl BioMart database (Ensembl genes 79, http://www.ensembl.org). Gene enrichment tests were implemented in the TopGO package from Bioconductor (http://www.bioconductor.org). In the tests, the number of occurrences for the tested and reference genes in one functional category was compared, and the comparisons were assessed based on a significance index using Fisher’s exact test. The total Ensembl-annotated coelacanth genes were used as the reference genes for all GO analyses in this study. Gene functional categories showing *p* < 0.01 were included as significantly enriched categories.

### Other analyses

All statistical tests were computed in R (http://www.R-project.org). The structure of the genes presented in Additional file 2: Figure S1 was constructed using fancyGENE [39]. The orthologies of the retrocopy-overlapped genes were searched using BioMart (http://www.ensembl.org) and BLAST+ [40].

### Availability of supporting data

The repeat-masked genome, the peptide sequences and the GO annotations data of the coelacanth are availability at Ensembl (http://www.ensembl.org). The RNA-seq data from tissues of the coelacanth, including gills, kidneys, pectoral fins, pelvic fins, pharynx, tail muscle and testis are availability at NCBI (http://www.ncbi.nlm.nih.gov) with SRA accession DRP000627 and SRX189186.

## Additional files

Additional file 1: Table S1. Informations of the retrocopies and their parent genes in the genome of Africa coelacanth. **Table S2.** Mapping informations about the mapping of retrocopies to Ensembl genes. **Table S3.** FPKMs of the retrocopies in each tissue. **Table S4.** Mean and median values of log(FPKM + 1), dS and dN/dS for the retrocopies expressed in different tissues. **Table S5.** Mean and median values of log(FPKM + 1), dS and dN/dS for the retrocopies uniquely expressed in different tissues. **Table S6.** The comparison of structure and expression informations between Coelacanth lineage-special retrocopy-overlapped genes and the rest of the retrocopy-overlapped genes. **Table S7.** Summary of over-represented GO terms for the retrocopy-overlapped genes that are lineage special to coelacanth. **Table S8.** List of the retrocopy-overlapped genes that specially lost in tetrapod. **Table S9.** Comparison of structure and expression informations between the retrocopy-overlapped genes that specially lost in tetrapod and those still exist. **Table S10.** Comparison of over-represented GO terms between the retrocopy-overlapped genes that specially lost in tetrapod and those that still exist in tetrapod. (XLS 485 kb) Additional file 2: Figure S1. Examples of retrocopy-overlapped genes. A) BRINP3 gene (ENSLACG00000008662), example of a gene overlapping with two retrocopies. B) esrrga gene (ENSLACG00000010017), example of a retrocopy-overlapped gene specifically lost in tetrapods. C) ENSLACG00000010078 gene, example of a coelacanth lineage-specific retrocopy-overlapped gene. Grey boxes represent exons, and dotted lines in genes represent introns. Black boxes represent retrocopies. The top-down order of each example: retrocopy-overlapped gene, retrocopy, and parent gene of the retrocopy. Genes are not drawn to a uniform scale. (DOC 93 kb)

## Abbreviations

dN: The number of nonsynonymous substitutions per non-synonymous site
dS: The number of synonymous substitutions per synonymous site
dN/dS: The ratio of dN to dS
LRT: Likelihood ratio test
FPKM: Fragments per kilobase of exon per million fragments mapped
GO: Gene ontology
ORF: Open reading frame
SRA: Sequence read archive
