# Supplementary material for: Evolutionary fate and implications of retrocopies in the African coelacanth genome
Source: BMC Genomics. 2015 Nov 10;16:915. doi: 10.1186/s12864-015-2178-9 (PMC4641402; doi:10.1186/s12864-015-2178-9)
Supplement: Additional file 2: Figure S1. — Examples of retrocopy-overlapped genes. A) BRINP3 gene (ENSLACG00000008662), example of a gene overlapping with two retrocopies. B) esrrga gene (ENSLACG00000010017), example of a retrocopy-overlapped gene specifically lost in tetrapods. C) ENSLACG00000010078 gene, example of a coelacanth lineage-specific retrocopy-overlapped gene. Grey boxes represent exons, and dotted lines in genes represent introns. Black boxes represent retrocopies. The top-down order of each example: retrocopy-overlapped gene, retrocopy, and parent gene of the retrocopy. Genes are not drawn to a uniform scale. (DOC 93 kb) [file 12864_2015_2178_MOESM2_ESM.doc]

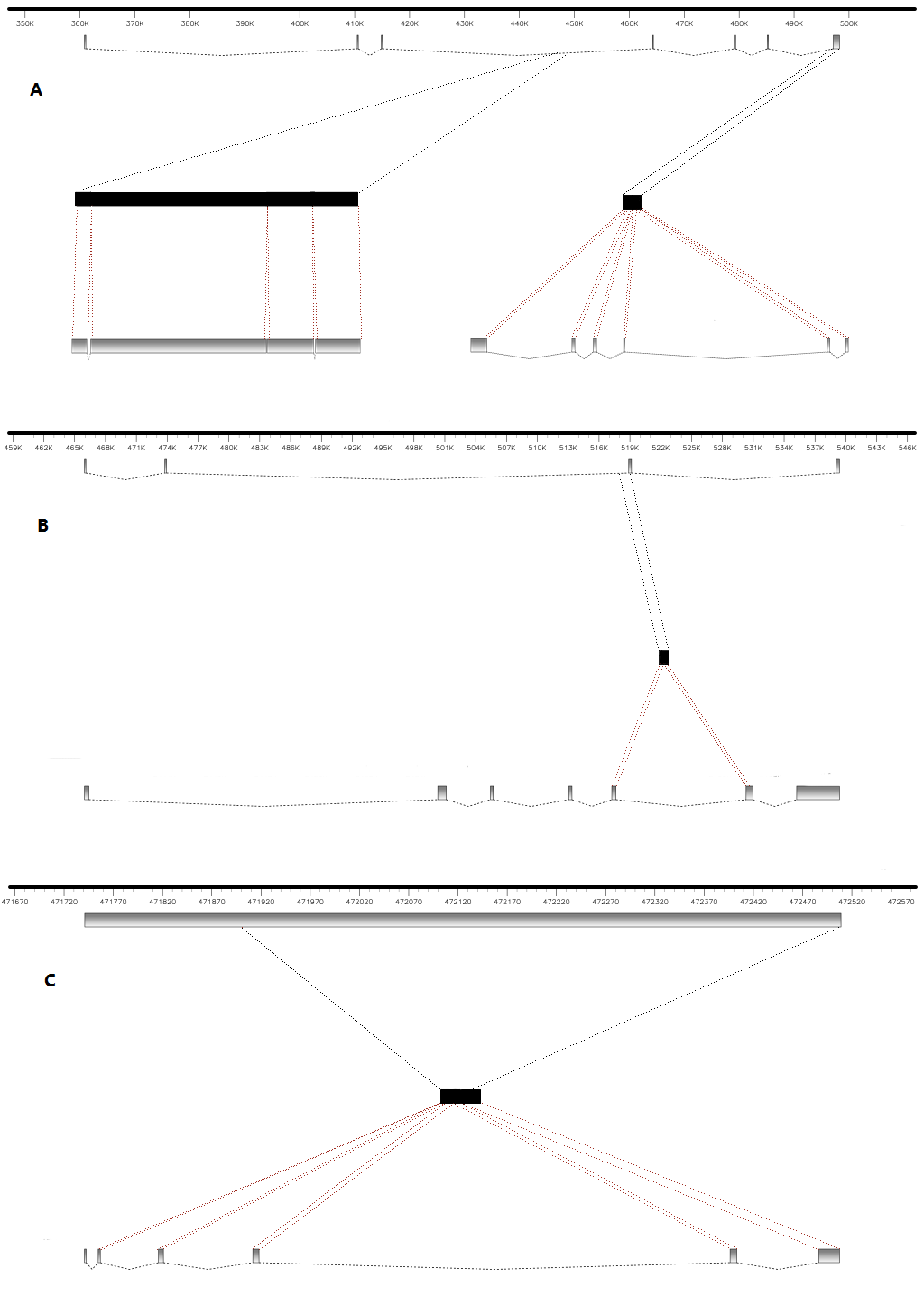


**Fig. S1.** Examples of retrocopy-overlapped genes. A) BRINP3 gene (ENSLACG00000008662), example of a gene overlapping with two retrocopies. B) esrrga gene (ENSLACG00000010017), example of a retrocopy-overlapped gene specifically lost in tetrapods. C) ENSLACG00000010078 gene, example of a coelacanth lineage-specific retrocopy-overlapped gene. Grey boxes represent exons, and dotted lines in genes represent introns. Black boxes represent retrocopies. The top[-](http://cn.bing.com/dict/search?q=-&FORM=BDVSP6&mkt=zh-cn)down [order](http://cn.bing.com/dict/search?q=order&FORM=BDVSP6&mkt=zh-cn) [o](http://cn.bing.com/dict/search?q=of&FORM=BDVSP6&mkt=zh-cn)f each example: retrocopy-overlapped gene, retrocopy, and parent gene of the retrocopy. Genes are not drawn to a uniform scale.
